# Time trends and inequalities of physical activity domains and sitting time in South America

**DOI:** 10.7189/jogh.12.04027

**Published:** 2022-04-02

**Authors:** André O Werneck, Raphael HO Araujo, Nicolas Aguilar-Farias, Gerson Ferrari, Javier Brazo-Sayavera, Christian García-Witulski, Victor Z Dourado, Luciana L Barboza, Ellen CM Silva, Kabir P Sadarangani, Ramfis Nieto-Martinez, Antonio García-Hermoso, Robinson Ramírez-Vélez, Danilo R Silva, South American Physical Activity and Sedentary Behavior Network (SAPASEN) collaborators

**Affiliations:** 1 Center for Epidemiological Research in Nutrition and Health, Department of Nutrition, School of Public Health, Universidade de São Paulo, São Paulo; Brazil; 2 Graduation Program in Health Sciences, Londrina State University, Londrina, Brazil; 3 Department of Physical Education, Sports and Recreation, Universidad de La Frontera, Temuco, Chile; 4 Escuela de Ciencias de la Actividad Física, el Deporte y la Salud, Universidad de Santiago de Chile (USACH), Santiago, Chile; 5 Department of Sports and Computer Science, Universidad Pablo de Olavide, Seville, Spain; 6 PDU EFISAL, Centro Universitario Regional Noreste, Universidad de la República, Rivera, Uruguay; 7 Facultad de Ciencias Económicas, Pontificia Universidad Católica Argentina, Buenos Aires, Argentina; 8 Universidad Espíritu Santo, Ecuador; 9 Department of Human Movement Sciences, Laboratory of Epidemiology and Human Movement (EPIMOV), Federal University of São Paulo, Santos, SP, Brazil; 10 Postgraduate Program in Physical Education, University of Brasília, Brasília, Brazil; 11 Postgraduate Program in Health Sciences, Londrina State University, Londrina, Brazil; 12 Escuela de Kinesiología, Facultad de Salud y Odontología, Universidad Diego Portales, Santiago, Chile; 13 Universidad Autónoma de Chile, Chile; 14 Department of Global Health and Population, Harvard TH Chan School of Public Health, Boston, Massachusetts, USA; 15 Foundation for Clinic, Public Health, and Epidemiology Research of Venezuela (FISPEVEN INC), Caracas, Venezuela; 16 LifeDoc Health, Memphis, Tennessee, USA; 17 Navarrabiomed, Hospital Universitario de Navarra (HUN), Navarra Institute for Health Research (IdiSNA), Universidad Pública de Navarra (UPNA), Pamplona, Spain; 18 CIBER of Frailty and Healthy Aging (CIBERFES), Instituto de Salud Carlos III, Madrid, Spain; 19 Facultad de Ciencias de la Educación, Unidad Central del Valle del Cauca (UCEVA), Tuluá, Valle del Cauca, Colombia

## Abstract

**Background:**

We aimed to investigate time trends and inequalities of different physical activity (PA) domains and sitting time (ST) in adults from South American countries.

**Methods:**

We included cross-sectional data of nationally representative surveys on adults (n = 597 843) from nine South American countries (Argentina, Bolivia, Brazil, Chile, Colombia, Ecuador, Peru, Uruguay, and Venezuela), with data collection time frames ranging from 2005 to 2020. Data on different PA domains (leisure-time, transport, and occupational) and ST were assessed through questionnaires. Trends according to education level (quintiles), gender (m/w), and age group (18-34 years, 35-49 years, 50-64 years) were estimated for the harmonized indicators of nonzero PA in the different domains, ≥150 min/week of total PA and ≥8 hours/d of ST.

**Results:**

Chile (2009/2010 = 78.9% vs 2016/2017 = 70.5%), and Peru (2009/2010 = 78.6% vs 2011 = 69.6%) reduced total PA, while Brazil (2013 = 57.3% vs 2019 = 67.0%) and Uruguay (2006 = 69.4% vs 2013 = 79.4%) increased, and Argentina and Venezuela maintained. There was an increasing trend for ST in Argentina, Peru, and Uruguay. Leisure-time PA increased in most countries (6/8 countries). Transport PA was relatively stable, while occupational PA presented mixed findings. Education inequalities increased over time for total and leisure-time PA, while age and gender inequalities were relatively constant.

**Conclusions:**

Future South American countries' efforts may be warranted to promote PA and reduce ST in adults, while addressing inequalities when implementing actions.

Physical inactivity and excessive sedentary behavior are recognized risk factors for different cardiovascular diseases, types of cancer, and mental disorders [1-4]. However, a high prevalence of both risk behaviors has been shown worldwide [5-7]. Data have shown that approximately 27.5% of the worldwide population of adults do not meet the recommendations for physical activity [5]. Also, adults spend around 4.7 hours/d in sedentary behavior [7]. This scenario is even more worrying in Latin America, which presents the highest values of physical inactivity, with approximately 40% of the adults not fulfilling the physical activity recommendations [5,8]. Also, substantial rates of elevated sedentary behavior have been reported in countries of that region [8].

The World Health Organization (WHO) launched the Global Action Plan on Physical Activity that aims to relatively reduce by 15% the global prevalence of insufficient physical activity among adolescents and adults by 2030 [9]. Notwithstanding the relevance of cross-sectional studies on the prevalence of physical inactivity and excessive sedentary behavior, the comprehension of temporal trends can contribute to the directions of priority areas for policymakers, as well as to the assessment of the country's performance in reducing physical inactivity and sedentary behavior over the time.

In this regard, there is relative stability in physical activity trends worldwide [5], while there is no previous evidence of multi-country investigations for sedentary behavior. However, in addition to the importance of general time trends of these behaviors over time, looking at the differences between population subgroups can evidence inequalities on the trends and help the guidance of public policies with a focus on those subgroups with less favorable figures. For instance, a recent study from Brazil revealed that leisure-time physical activity increased between 2008 and 2019, however, the educational, gender, age, and type of residency inequalities also increased, with a lower increase among people with lower education, women, older adults, and residing in rural areas, respectively [10]. This is especially important when considering the different domains of physical activity, given that leisure-time physical activity usually shows elevated inequalities [8].

South American countries present distinct characteristics. With an accelerated urbanization process throughout the 20th century, this region became the most urbanized worldwide, but also the most unequal one [11]. Therefore, if policy makers and decision takers continue to plan, invest, and implement as they are doing, the inequalities in the practice and trends in different physical activity domains can be even more pronounced, in opposition to the United Nations Sustainable Development Goals [12]. However, the time trends and inequalities of physical activity domains and sitting time in other countries of South America are unknown. Therefore, we aimed to investigate time trends and inequalities of different physical activity domains and sitting time in adults from South American countries using nationally representative data.

## METHODS

### Design

This is a cross-sectional, multi-country study conducted by the South American Physical Activity and Sedentary Behavior Network (SAPASEN). The SAPASEN aims to harmonize national representative data sets with physical activity and sedentary behavior indicators from South American countries [13]. After the first analyses [6,8], we identified nine countries with more than one survey along the time (from 2005-2019) with physical activity and/or sitting time and included data from Argentina, Bolivia, Brazil, Chile, Colombia, Ecuador, Peru, Uruguay, and Venezuela.

### Sample

We analyzed data from the following countries: Argentina (2005, 2009, 2013 and 2018), Bolivia (2008 and 2016), Brazil (2008, 2015, 2013, and 2019), Chile (2009-2010 and 2016-2017), Colombia (2005, 2010 and 2015), Ecuador (2011-2012 and 2018), Peru (2007-2008, 2009-2010 and 2011), Uruguay (2006 and 2013), and Venezuela (2014-2017 and 2018-2020*)*. More information on the surveys can be found in Table S1 in the **Online Supplementary Document**.

Data of each country were pooled, excluding young and older people, including the age range between 18 and 64 years. The exceptions were the survey from Ecuador during 2011-2012, which only included younger than 60 years, both studies from Bolivia that included participants between 18 and 49 years and Uruguay's 2006 STEPS survey that included participants between 25 and 64 years. All samples were calculated through complex sampling, with several levels. More detailed sampling methodology can be found in Table S1 in the **Online Supplementary Document**.

After excluding participants older than 64 and younger than 18 years as well as missing data, the final sample was composed of 597 843 adults (more information on the sample size and missing data from each survey can be found in Table S1 in the **Online Supplementary Document**). Specific sampling weights originally calculated from each survey aiming the extrapolation of data for population characteristics representativity were used in the analyzes.

### Physical activity and sitting time

The International Physical Activity Questionnaire (IPAQ) [14] was used in the surveys from Argentina, Colombia, Ecuador (2011-2012) Peru, and Venezuela, while the Global Physical Activity Questionnaire (GPAQ) [15] was used on surveys from Chile, Ecuador (2018) and Uruguay. Brazil used a specific questionnaire derived from another survey (Surveillance System for Risk and Protective Factors for Chronic Diseases by Telephone Survey – VIGITEL) [16]. There were questions regarding leisure-time, transportation, and occupational physical activity in all questionnaires. Bolivia included a specific question regarding leisure-time physical activity and another for sitting time. Argentina had the short version of the IPAQ but included questions about physical activity practice in each domain. The 2008 and 2015 surveys from Brazil, and the 2007-2008 survey from Peru only included questions regarding leisure-time physical activity. The surveys from Colombia and Ecuador (2011-2012) included questions regarding leisure-time and transport. Total sitting time (including leisure time, occupational and transport) was the indicator of sitting time in all the included surveys. The IPAQ, GPAQ and VIGITEL are validated questionnaires, while the specific questions used in Bolivia and Peru (2007-2008) were not previously validated [14-16].

We used as indicators the nonzero practice of physical activity during leisure-time, transport, and occupational domains considering our aim of estimating the practice of each domain as well as to increase the comparability among the surveys. The sum of the physical activity domains (leisure-time, transport and occupational) was used as an indicator of total physical activity, and we classified as physically active those who reported more than 150 min/week [17]. Sitting time was classified using the cutoff point of 8 hours/d [18]. More information about the specific questionnaires is presented in Table S1 in the **Online Supplementary Document**.

### Indicators of socioeconomic inequalities

Gender (men and women), chronological age (18-34 years, 35-49 years, 50-64 years), and educational level (quintiles or categories) were considered as sociodemographic indicators. We classified educational level into categories based on the years of education, or the highest level of education reached by the individuals in each survey. More information is presented in Table S1 in the **Online Supplementary Document**.

### Statistics

Eight countries were included in the analysis for leisure-time physical activity, while seven countries were included in the analysis of transport, six in the analysis of total physical activity, five in the analysis of occupational physical activity, and six in the analysis of sitting time. We included five surveys from Argentina, four from Brazil, three from Colombia and Peru, and two from Bolivia, Chile, Ecuador, and Uruguay. We used values of frequency and 95% confidence interval to estimate the prevalence of each physical activity domain, total physical activity and sitting time according to each subgroup in every survey. We defined the gap as the absolute difference between the quintiles 5 and 1 of education, between the men and women, as well as between the 18-34 and 50-64 groups of age. All the analyses were conducted using the Stata 15.1 software.

## RESULTS

**Figure 1:**
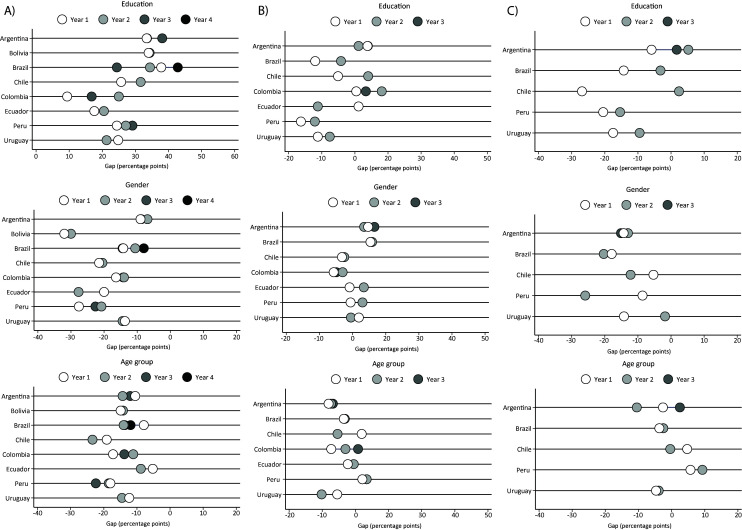
Inequalities over the years of physical activity domains according to education (highest quintile – lowest quintile), gender (women – men) and age group (50-64 – 18-34) in percentage points. Argentina: year 1 = 2005, year 2 = 2009, year 3 = 2013, year 4 = 2018. Bolivia: year 1 = 2008, year 2 = 2016. Brazil: year 1 = 2008, year 2 = 2013, year 3 = 2015, year 4 = 2019. Chile: year 1 = 2009-2010, year 2 = 2016-2017. Colombia: year 1 = 2005, year 2 = 2010, year 3 = 2015. Ecuador: year 1 = 2011-2012, year 2 = 2018. Peru: year 1 = 2007-2008, year 2 = 2009-2010, year 3 = 2011. Uruguay: year 1 = 2006, year 2 = 2013. **Panel A.** Leisure-time physical activity (nonzero). **Panel B.** Transport physical activity (nonzero). **Panel C**. Occupational physical activity (nonzero).

The temporal trends in leisure-time physical activity according to education, gender and age are presented in **Table 1**. Except for Colombia and Ecuador, leisure-time physical activity increased in all countries, with Brazil (2008 = 27.2% vs 2019 = 42.9%) and Peru (2007/2008 = 24.0% vs 2011 = 40.4%) presenting the largest increases. However, the increase in leisure-time physical activity was not similar among the population groups and the difference between the first and the fifth quintiles of education increased, especially in the highest quintiles of education (**Figure 1**). Although men and younger adults presented a higher prevalence of leisure-time physical activity, the gender and age inequalities were similar across the surveys, with minor increases for gender inequalities in Chile and Ecuador and age inequalities in Brazil, Chile, Ecuador, and Peru (**Figure 1**).

**Table 1:** Temporal trends of nonzero leisure-time physical activity practice in South American countries*

	Total	Quintiles of education						Gender			Age group			
	**Q1**	**Q2**	**Q3**	**Q4**	**Q5**	**Gap**	**Men**	**Women**	**Gap**	**18-34**	**35-49**	**50-64**	**Gap**
Argentina														
2009	40.5 (39.5-41.4)	21.3 (18.7-24.1)	28.6 (26.7-30.5)	37.0 (34.9-39.2)	41.9 (40.0-43.8)	54.7 (53.0-56.4)	33.4	45.2 (43.7-46.6)	36.2 (35.1-37.4)	-9.0	45.5 (44.1-47.0)	37.4 (35.9-39.0)	35.0 (33.2-36.8)	-10.5
2013	43.0 (41.9-44.1)	25.1 (22.2-28.2)	27.9 (25.8-30.1)	42.9 (40.2-45.7)	45.3 (43.1-47.6)	58.6 (56.6-60.6)	33.5	46.6 (45.0-48.3)	39.7 (38.3-41.1)	-6.9	50.0 (48.1-51.9)	42.4 (40.3-44.4)	35.7 (34.0-37.4)	-14.3
2018	46.5 (45.3-47.6)	21.5 (17.9-25.6)	29.6 (26.9-32.4)	40.5 (27.8-43.2)	47.5 (45.1-49.8)	59.6 (57.8-61.4)	38.1	51.1 (49.3-52.8)	42.3 (40.8-43.8)	-8.8	51.8 (49.9-53.7)	44.1 (42.2-46.0)	39.8 (37.7-42.0)	-12.0
Bolivia†														
2008	30.9 (30.1-31.7)	13.8 (12.8-14.8)	26.9 (23.8-30.3)	40.6 (38.3-43.0)	40.6 (38.7-42.5)	47.8 (45.9-49.6)	34.0	55.6 (53.8-57.4)	23.6 (22.8-24.4)	-32.0	36.6 (35.5-37.7)	21.7 (20.5-22.9)	-	-14.9
2016	37.8 (36.7-38.7)	13.2 (10.1-17.1)	25.2 (23.3-27.1)	39.0 (37.5-40.6)	46.9 (43.4-50.4)	47.5 (45.3-49.6)	34.3	60.1 (58.1-62.1)	30.1 (29.0-31.2)	-30.0	43.3 (42.0-44.6)	29.1 (27.6-30.6)	-	-14.2
Brazil														
2008	27.2 (26.9-27.5)	10.0 (9.4-10.5)	17.5 (17.2-17.9)	28.1 (27.5-28.7)	34.4 (33.9-35.0)	47.8 (47.0-48.6)	37.8	35.9 (35.5-36.4)	21.7 (21.4-22.0)	-14.2	31.4 (31.0-31.8)	24.5 (24.1-24.9)	23.5 (23.0-24.1)	-7.9
2013	32.0 (31.3-32.8)	14.9 (13.4-16.6)	19.9 (18.6-21.2)	31.4 (29.6-33.2)	36.7 (35.3-38.1)	49.3 (47.5-51.0)	34.4	37.6 (36.5-38.7)	27.0 (26.1-27.9)	-10.6	39.2 (38.0-40.4)	27.5 (26.4-28.6)	25.2 (23.9-26.7)	-14.0
2015	24.1 (23.7-24.6)	10.5 (9.4-11.8)	15.9 (15.2-16.6)	23.3 (22.3-24.3)	27.5 (26.7-28.3)	34.9 (33.9-36.0)	24.4	31.6 (31.0-32.3)	17.2 (16.7-17.7)	-14.4	30.9 (30.1-31.6)	20.9 (20.2-21.6)	17.2 (16.5-18.0)	-13.7
2019	42.9 (42.3-43.6)	19.4 (17.0-22.0)	27.5 (26.4-28.6)	37.6 (36.0-39.2)	46.3 (45.1-47.5)	62.2 (60.8-63.5)	42.8	47.1 (46.2-48.1)	39.1 (38.2-40.0)	-8.0	48.8 (47.6-49.9)	41.2 (40.1-42.2)	36.9 (35.7-38.0)	-11.9
Chile														
2009/10	33.4 (30.9-36.1)	18.6 (14.3-24.0)	27.6 (23.3-32.3)	34.8 (30.4-39.5)	45.2 (36.2-54.5)	44.3 (37.4-51.5)	25.7	44.4 (40.3-48.6)	22.9 (20.2-25.9)	-21.5	43.5 (39.2-47.8)	29.0 (24.8-33.5)	24.4 (20.2-29.1)	-19.1
2016/17	31.4 (29.0-34.0)	13.7 (8.9-20.5)	23.3 (19.4-27.8)	30.2 (26.1-34.7)	43.6 (35.0-52.7)	45.3 (39.3-51.5)	31.6	41.8 (37.8-45.9)	21.3 (18.6-24.2)	-20.5	43.1 (38.7-47.6)	28.3 (24.2-32.7)	19.7 (16.0-24.0)	-23.4
Colombia														
2005	43.6 (42.2-45.0)	40.9 (37.4-44.5)	41.1 (37.4-44.9)	42.3 (39.2-45.3)	45.3 (42.0-48.6)	50.3 (47.3-53.2)	9.4	52.7 (50.5-54.9)	36.3 (34.5-38.1)	-16.4	52.1 (50.1-54.1)	35.9 (33.4-38.5)	34.9 (31.8-38.1)	-17.2
2010	41.0 (40.0-42.0)	23.7 (18.9-29.1)	32.0 (30.2-33.9)	42.1 (40.7-43.6)	49.4 (46.0-52.9)	48.7 (46.0-51.4)	25.0	49.1 (47.5-50.7)	35.1 (33.8-36.5)	-14.0	47.2 (45.7-48.8)	35.9 (34.2-37.7)	36.1 (33.9-38.2)	-11.1
2015	36.5 (35.3-37.8)	26.4 (24.3-28.6)	31.7 (28.9-34.6)	36.0 (33.4-38.7)	37.9 (35.6-40.2)	43.2 (40.2-46.2)	16.8	44.1 (42.3-45.9)	29.9 (28.2-31.7)	-14.2	43.9 (42.1-45.8)	30.5 (28.2-32.9)	30.1 (27.7-32.6)	-13.8
Ecuador‡§														
2011/12	36.2 (35.1-37.3)	26.5 (24.7-28.4)	35.1 (32.3-38.0)	39.3 (37.5-41.2)	43.2 (37.6-49.1)	44.1 (41.5-46.7)	17.6	47.5 (45.8-49.2)	25.7 (24.4-27.0)	-20.0	41.6 (40.1-43.0)	32.0 (30.4-33.7)	26.8 (23.6-30.3)	-5.2
2018	37.8 (36.1-39.6)	25.1 (22.6-27.8)	29.4 (22.1-37.9)	44.3 (41.3-47.3)	51.0 (41.3-60.5)	45.6 (41.5-49.7)	20.5	52.0 (49.2-54.7)	24.4 (22.3-26.5)	-27.7	48.8 (45.9-51.6)	33.4 (30.6-36.3)	24.6 (21.5-28.0)	-8.8
Peru														
2007/08	24.0 (23.0-25.0)	9.3 (8.0-11.0)	20.1 (18.4-21.9)	28.6 (26.6-30.7)	29.2 (26.4-30.1)	33.7 (30.7-36.9)	24.4	38.9 (37.2-40.6)	11.3 (10.3-12.4)	-27.6	31.6 (30.0-33.2)	19.7 (18.1-21.4)	13.6 (11.8-15.6)	-18.0
2009/10	39.7 (38.8-40.6)	23.8 (22.3-25.4)	35.6 (34.0-37.3)	43.9 (42.0-45.7)	48.3 (45.8-50.8)	50.9 (48.4-53.5)	27.1	51.6 (50.3-53.0)	30.8 (29.6-31.9)	-20.8	48.2 (46.8-49.6)	36.6 (35.2-38.2)	29.7 (28.0-31.5)	-18.5
2011	40.4 (39.0-41.7)	24.7 (22.3-27.2)	33.7 (31.4-36.1)	44.6 (41.8-47.3)	48.4 (44.8-52.1)	53.8 (50.1-57.5)	29.1	53.1 (51.1-55.1)	30.5 (28.8-32.2)	-22.6	52.1 (50.0-54.3)	34.8 (32.6-37.0)	29.7 (27.2-32.4)	-22.4
Uruguay§														
2006	31.4 (29.1-33.8)	21.7 (16.1-28.6)	24.8 (20.8-29.3)	26.7 (22.5-31.5)	36.4 (30.9-42.2)	46.5 (40.9-52.2)	24.8	38.6 (34.6-42.6)	24.9 (22.5-27.4)	-13.7	38.9 (34.1-43.9)	29.2 (25.7-33.1)	26.6 (23.3-30.0)	-12.3
2013	44.8 (42.4-47.2)	30.8 (26.1-36.0)	37.9 (33.3-42.7)	50.0 (45.3-54.1)	57.9 (52.2-63.5)	52.1 (42.3-61.7)	21.3	52.2 (48.4-56.0)	37.8 (35.0-40.8)	-14.4	51.8 (47.7-55.8)	40.2 (36.2-44.2)	37.3 (33.5-41.3)	-14.5

*Values are presented in percentage (95% CI) and the gap is presented as percentage point. Gap of education refers to Q5- Q1. Gap of gender refers to Women – Men. Gap of age group refers to 50-64 – 18-34 or 35-49 – 18-34 for Bolivia.

†Bolivia only included people between 18 and 49 years.

‡Ecuador 2011/12 only included adults between 18 and 59 years.

§Uruguay 2006 only included adults between 25 and 64 years.

**Table 2** shows the trends and inequalities of transport physical activity. There was an increase in transport physical activity in Argentina (2009 = 57.3% vs 2018 = 65.9%) and Peru (2009/2010 = 46.3% vs 2011 = 70.5%), while the prevalence of transport physical activity was maintained in Brazil, Chile, Colombia, and Uruguay and reduced in Ecuador. The inequalities regarding education, gender and age group were minor and similar across the surveys (**Figure 1**).

**Table 2:** Temporal trends of nonzero transport physical activity practice in South American countries*

	Total	Quintiles of education						Gender			Age group			
	**Q1**	**Q2**	**Q3**	**Q4**	**Q5**	**Gap**	**Men**	**Women**	**Gap**	**18-34**	**35-49**	**50-64**	**Gap**
Argentina														
2009	57.3 (56.3-58.2)	54.5 (51.4-57.6)	55.4 (53.3-57.5)	60.0 (57.8-62.1)	56.2 (54.3-58.1)	58.3 (56.6-60.0)	3.8	54.8 (53.3-56.2)	59.5 (58.3-60.7)	4.7	60.8 (59.3-62.2)	55.7 (54.1-57.3)	52.7 (50.9-54.5)	-8.1
2013	49.3 (48.2-50.4)	49.4 (46.0-52.9)	46.1 (43.7-48.6)	51.3 (48.5-54.0)	49.1 (46.8-51.4)	50.5 (48.6-52.5)	1.1	47.4 (45.7-49.0)	50.9 (49.5-52.4)	3.5	53.4 (51.5-55.2)	47.5 (45.4-49.6)	46.0 (44.2-47.8)	-7.4
2018	65.9 (64.8-67.0)	63.9 (58.9-68.6)	61.4 (58.4-64.3)	67.9 (65.5-70.3)	64.9 (62.6-67.0)	67.8 (66.1-69.5)	3.9	62.5 (60.8-64.1)	69.1 (67.7-70.4)	6.6	70.1 (68.4-71.8)	62.2 (60.3-64.0)	63.3 (61.2-65.4)	-6.8
Brazil														
2013	51.4 (50.7-52.2)	54.6 (52.4-56.8)	56.2 (54.7-57.8)	53.5 (51.6-55.4)	51.0 (49.6-52.5)	42.6 (40.9-44.4)	-12.0	48.6 (47.5-49.8)	54.0 (53.0-55.0)	5.4	52.8 (51.6-54.0)	51.2 (50.0-52.5)	49.3 (47.7-50.9)	-3.5
2019	51.3 (50.6-51.9)	49.0 (45.9-52.1)	54.3 (53.0-55.5)	52.9 (51.2-54.5)	52.9 (51.7-54.1)	44.8 (43.4-46.2)	-4.2	48.2 (47.2-49.1)	54.1 (53.2-55.0)	5.9	53.3 (52.1-54.4)	50.0 (48.9-51.0)	50.1 (48.9-51.3)	-3.2
Chile														
2009/10	66.2 (63.7-68.7)	65.4 (59.6-70.8)	69.9 (65.2-74.2)	67.4 (63.1-71.4)	64.7 (54.5-73.7)	60.3 (53.1-67.2)	-5.1	67.9 (63.8-71.7)	64.7 (61.4-67.8)	-3.2	67.6 (63.4-71.5)	62.8 (58.1-67.2)	69.5 (65.2-73.4)	1.9
2016/17	67.9 (65.3-70.3)	67.0 (60.0-73.3)	65.8 (60.8-70.5)	64.5 (59.8-69.0)	77.2 (69.1-83.6)	71.0 (65.6-75.8)	4.0	69.1 (65.2-72.7)	66.6 (63.3-69.8)	-2.5	72.3 (68.3-76.1)	63.3 (58.6-67.7)	66.9 (62.4-71.2)	-5.4
Colombia														
2005	75.1 (73.9-76.2)	76.4 (73.5-79.0)	72.3 (68.8-75.5)	73.2 (70.6-75.6)	79.0 (76.5-81.4)	76.8 (74.3-79.1)	0.4	78.2 (76.4-79.9)	72.5 (70.9-74.1)	-5.7	77.7 (76.1-79.1)	73.9 (71.7-76.0)	70.4 (67.2-73.4)	-7.3
2010	78.0 (77.1-78.8)	68.9 (63.3-74.0)	76.1 (74.4-77.8)	79.7 (78.4-80.9)	78.2 (75.2-80.9)	77.0 (74.7-79.2)	8.1	79.7 (78.4-80.9)	76.7 (75.6-77.9)	-3.0	79.2 (77.9-80.4)	77.5 (75.9-78.9)	76.2 (74.3-78.1)	-3.0
2015	76.6 (75.5-77.6)	73.9 (71.5-76.1)	78.6 (76.1-80.8)	77.0 (74.7-79.2)	76.5 (74.5-78.4)	77.2 (74.7-79.4)	3.3	79.2 (77.8-80.6)	74.3 (72.8-75.8)	-4.9	76.1 (74.5-77.6)	77.2 (75.2-79.1)	76.9 (74.7-78.9)	0.8
Ecuador†														
2011/12	78.2 (77.2-79.1)	78.0 (76.2-79.7)	78.0 (75.2-80.5)	78.6 (77.1-80.1)	81.5 (76.6-85.5)	76.9 (74.6-79.0)	1.1	78.6 (77.2-80.0)	77.7 (76.5-79.0)	-0.9	78.8 (77.6-80.0)	77.9 (76.4-79.4)	76.5 (73.1-79.7)	-2.3
2018	72.7 (71.0-74.3)	76.7 (74.1-79.1)	80.1 (72.4-86.0)	73.0 (70.1-75.7)	71.0 (61.0-79.3)	65.5 (61.5-69.4)	-11.2	71.0 (68.4-73.4)	74.4 (72.2-76.4)	3.4	73.9 (71.3-76.4)	69.7 (66.8-72.5)	74.4 (70.9-77.6)	-0.5
Peru														
2009/10	46.3 (45.4-47.2)	57.1 (55.3-58.9)	49.3 (47.6-51.1)	42.0 (40.2-43.9)	40.2 (37.8-42.6)	40.8 (38.4-43.3)	-16.3	46.6 (45.3-48.0)	46.0 (44.8-47.2)	-0.6	45.2 (43.8-46.6)	46.9 (45.4-48.4)	47.3 (45.4-49.1)	2.1
2011	70.5 (69.2-71.7)	76.6 (74.2-78.8)	73.8 (71.6-75.9)	69.7 (67.0-72.2)	65.7 (62.1-69.1)	64.5 (60.8-68.0)	-12.1	68.8 (66.9-70.7)	71.8 (70.1-73.3)	3.0	68.9 (66.9-70.9)	70.9 (68.8-72.9)	72.3 (69.8-74.7)	3.4
Uruguay‡														
2006	63.3 (61.0-65.6)	71.8 (65.3-77.5)	66.3 (61.8-70.6)	60.8 (56.0-65.5)	60.8 (55.0-66.3)	60.6 (54.9-66.0)	-11.2	62.3 (58.3-66.2)	64.2 (61.5-66.9)	1.9	68.6 (63.9-72.9)	59.4 (55.5-63.3)	63.1 (59.4-66.6)	-5.5
2013	60.5 (58.2-62.9)	58.1 (52.9-63.2)	63.0 (58.3-67.5)	60.8 (56.5-64.9)	62.0 (56.3-67.4)	50.5 (40.7-60.2)	-7.6	60.8 (57.0-54.4)	60.3 (57.4-63.2)	-0.5	66.5 (62.6-70.1)	54.9 (50.8-58.9)	56.3 (52.3-60.2)	-10.2

*Values are presented in percentage (95% CI) and the gap is presented as percentage point. Gap of education refers to Q5- Q1. Gap of gender refers to Women – Men. Gap of age group refers to 50-64 – 18-34.

†Ecuador 2011/12 only included adults between 18 and 59 years.

‡Uruguay 2006 only included adults between 25 and 64 years.

The trends of occupational physical activity and inequalities are presented in **Table 3**. While the prevalence of occupational physical activity increased in Argentina, Brazil, and Uruguay, it decreased in Chile and Peru. The inequalities regarding education quintiles were reduced among all the countries, while the gender inequalities increased in all countries (except Uruguay), and inequalities regarding age groups were maintained over the years (**Figure 1**).

**Table 3:** Temporal trends of nonzero occupational physical activity practice in South American countries

	Total	Quintiles of education						Gender			Age group			
	**Q1**	**Q2**	**Q3**	**Q4**	**Q5**	**Gap**	**Men**	**Women**	**Gap**	**18-34**	**35-49**	**50-64**	**Gap**
Argentina														
2009	29.8 (28.9-30.7)	31.3 (28.4-34.3)	31.7 (29.8-33.7)	32.9 (30.8-35.1)	30.6 (28.8-32.5)	25.4 (24.0-26.9)	-5.9	37.3 (35.9-38.7)	23.0 (22.0-24.1)	-14.3	29.5 (28.2-30.9)	32.6 (31.1-34.1)	26.9 (25.3-28.5)	-2.6
2013	25.8 (24.8-26.8)	20.5 (18.0-23.4)	25.1 (23.0-27.4)	25.7 (23.4-28.1)	28.5 (26.5-30.6)	25.7 (24.1-27.5)	5.2	32.6 (31.1-34.2)	19.6 (18.5-20.8)	-13.0	28.6 (27.0-30.3)	31.8 (30.0-33.8)	18.1 (16.6-19.5)	-10.5
2018	35.0 (33.9-36.1)	31.6 (27.1-36.5)	33.7 (31.0-36.6)	37.0 (34.4-39.7)	37.2 (35.0-39.5)	33.3 (31.5-35.0)	1.7	42.9 (41.1-44.6)	27.8 (26.5-29.2)	-15.1	31.9 (30.2-33.7)	39.7 (37.8-41.5)	34.4 (32.3-36.5)	2.5
Brazil														
2013	18.4 (17.8-19.0)	22.5 (20.8-24.4)	26.1 (24.8-27.5)	21.3 (19.7-22.9)	15.9 (14.9-17.0)	8.2 (7.3-9.1)	-14.3	27.8 (26.8-28.9)	9.9 (9.3-10.5)	-17.9	18.7 (17.8-19.7)	20.5 (19.5-21.6)	15.0 (13.9-16.2)	-3.7
2019	33.6 (32.9-34.2)	28.1 (25.2-31.3)	38.2 (37.0-39.5)	37.3 (35.6-39.0)	34.8 (33.6-36.0)	24.9 (23.7-26.1)	-3.2	44.2 (43.2-45.2)	23.9 (23.1-24.7)	-20.3	31.9 (30.8-33.0)	38.5 (37.5-39.6)	29.4 (28.3-30.6)	-2.5
Chile														
2009/10	60.6 (58.0-63.1)	65.6 (59.6-71.1)	68.5 (64.0-72.7)	64.9 (60.6-69.0)	56.6 (47.8-65.0)	38.7 (32.3-45.5)	-26.9	63.3 (59.3-67.1)	58.0 (54.7-61.3)	-5.3	57.2 (52.9-61.4)	63.3 (58.8-67.5)	61.9 (57.2-66.3)	4.7
2016/17	42.5 (39.9-45.2)	36.8 (30.1-44.1)	45.0 (40.1-50.1)	46.6 (41.9-51.4)	37.1 (28.7-46.3)	39.2 (33.3-45.5)	2.4	48.7 (44.6-52.8)	36.5 (33.1-40.0)	-12.2	43.1 (38.7-47.7)	41.7 (37.0-46.5)	42.7 (38.1-47.4)	-0.4
Peru														
2009/10	64.7 (63.8-65.6)	75.9 (74.2-77.6)	72.4 (70.6-74.0)	57.8 (55.8-59.7)	59.0 (56.4-61.5)	55.4 (42.8-57.9)	-20.5	69.6 (68.2-71.0)	61.0 (60.0-62.3)	-8.6	60.0 (58.5-61.4)	69.6 (68.0-71.1)	65.7 (63.8-67.6)	5.7
2011	50.6 (49.2-51.9)	58.8 (56.0-61.6)	55.6 (53.1-58.0)	46.8 (44.0-49.6)	46.5 (42.9-50.2)	43.4 (39.8-47.1)	-15.4	65.2 (63.1-67.2)	39.3 (37.6-41.0)	-25.9	44.0 (41.9-46.1)	56.0 (53.8-58.3)	53.3 (50.6-56.1)	9.3
Uruguay†														
2006	35.8 (33.5-38.3)	40.7 (33.9-47.8)	42.8 (38.1-47.7)	37.8 (33.1-42.8)	34.7 (29.3-50.5)	23.2 (18.7-28.5)	-17.5	43.3 (39.2-47.4)	29.1 (26.6-31.7)	-14.2	36.7 (32.0-41.7)	38.1 (34.2-42.1)	32.0 (28.6-35.7)	-4.7
2013	50.7 (48.2-53.1)	51.5 (46.3-56.8)	57.3 (52.5-62.0)	50.0 (45.6-54.4)	43.4 (37.8-49.1)	42.0 (32.5-52.0)	-9.5	51.6 (47.7-55.4)	49.8 (46.8-52.8)	-1.8	51.1 (47.0-55.1)	52.9 (48.8-56.9)	47.2 (43.2-51.2)	-3.9

*Values are presented in percentage (95% CI) and the gap is presented as percentage point. Gap of education refers to Q5-Q1. Gap of gender refers to Women – Men. Gap of age group refers to 50-64 – 18-34.

†Uruguay 2006 only included adults between 25 and 64 years.

**Figure 2:**
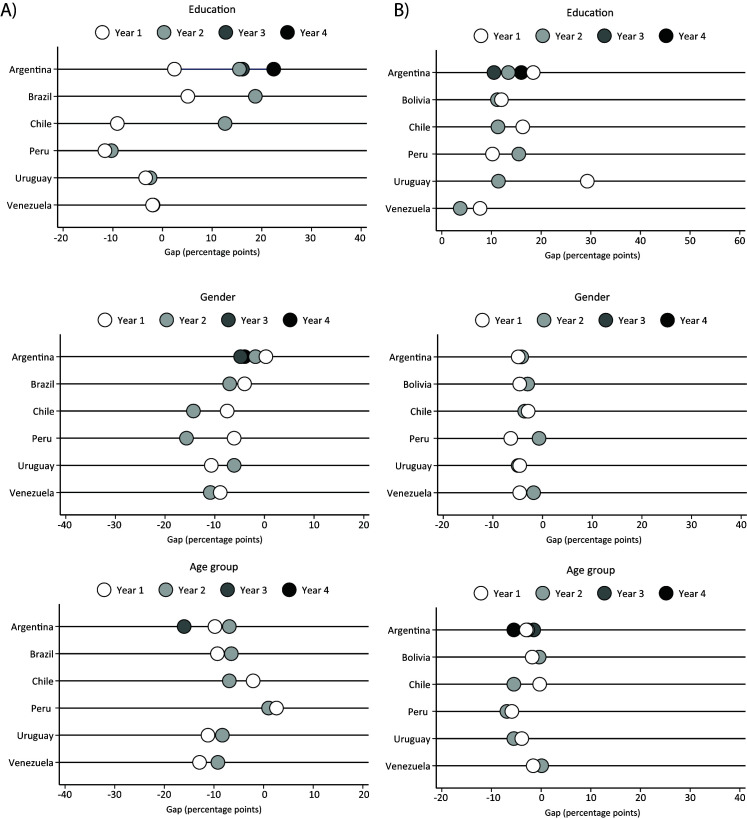
Inequalities over the years of total physical activity and sitting time according to education (highest quintile – lowest quintile), gender (women – men) and age group (50-64 – 18-34) in percentage points. **Panel A**. Total physical activity (at least 150 min/week). **Panel B**. Sitting time (at least 8 hours/d). Argentina: year 1 = 2005, year 2 = 2009, year 3 = 2013, year 4 = 2018. Bolivia: year 1 = 2008, year 2 = 2016. Brazil: year 1 = 2013, year 2 = 2019. Chile: year 1 = 2009-2010, year 2 = 2016-2017. Peru: year 1 = 2007-2008, year 2 = 2009-2010, year 3 = 2011. Uruguay: year 1 = 2006, year 2 = 2013.

There were mixed findings for the time trends of total physical activity (**Table 4**), with a decreasing trend in Chile (2009/2010 = 78.9% vs 2016/2017 = 70.5%) and Peru (2009/2010 = 78.6% vs 2011 = 69.6%), an increasing trend in Brazil (2013 = 57.3% vs 2019 = 67.0%), and Uruguay (2006 = 69.4% vs 2013 = 79.4%), and maintenance in Argentina and Venezuela (**Table 5**). There was an increase in the difference between the first and fifth quintile of education in Argentina (2005 = 2.4 p.p. vs 2018 = 22.4 p.p.), Brazil (2013 = 5.1 p.p. vs 2019 = 18.7 p.p.), Chile (2009/2010 = -9.1 p.p. vs 2016/2017 = 12.6 p.p.) and Colombia (2005 = -0.9 p.p. vs 2015 = 5.0 p.p.) (**Figure 2**). Also, there was increasing gender inequality in Argentina, Chile, and Peru, while the age inequalities were constant over time.

**Table 4:** Temporal trends of total physical activity (≥150 min/week) in South American countries

	Total	Quintiles of education						Gender			Age group			
	**Q1**	**Q2**	**Q3**	**Q4**	**Q5**	**Gap**	**Men**	**Women**	**Gap**	**18-34**	**35-49**	**50-64**	**Gap**
Argentina														
2005	70.6 (69.5-71.6)	69.1 (65.5-72.5)	68.7 (66.1-71.1)	73.9 (71.4-76.2)	69.3 (66.7-71.8)	71.5 (69.6-73.3)	2.4	70.4 (68.7-72.1)	70.7 (69.2-72.1)	0.3	74.5 (72.9-76.1)	69.5 (67.5-71.3)	64.7 (62.3-67.0)	-9.8
2009	70.9 (70.0-71.7)	61.1 (58.1-64.1)	65.3 (63.3-67.3)	71.3 (69.3-73.2)	71.3 (69.5-73.0)	76.6 (75.1-78.0)	15.5	71.8 (70.6-73.1)	70.0 (68.9-71.1)	-1.8	74.3 (73.0-75.5)	68.6 (67.1-70.1)	67.4 (65.7-69.1)	-6.9
2013	56.4 (55.3-57.5)	47.0 (43.5-50.4)	50.2 (47.8-52.7)	57.4 (54.7-60.1)	57.2 (54.9-59.4)	63.1 (61.1-65.1)	16.1	58.9 (57.3-60.6)	54.1 (52.7-55.6)	-4.8	63.6 (61.8-65.4)	57.4 (55.3-59.5)	47.6 (45.9-49.4)	-16.0
2018	68.1 (67.0-69.2)	53.4 (48.1-58.6)	59.0 (55.9-62.0)	64.9 (62.1-67.5)	68.1 (65.9-70.2)	75.8 (74.3-77.3)	22.4	70.2 (68.6-71.8)	66.2 (64.7-67.6)	-4.0	72.4 (70.7-74.1)	66.3 (64.4-68.1)	62.6 (60.4-64.7)	-9.8
Brazil														
2013	57.3 (56.5-58.0)	52.2 (50.0-54.4)	57.6 (56.1-59.1)	58.7 (56.8-60.5)	57.9 (56.5-59.3)	57.3 (55.6-59.1)	5.1	59.4 (58.2-60.5)	55.4 (54.4-56.4)	-4.0	60.5 (59.4-61.6)	57.4 (56.2-58.7)	51.2 (49.6-52.8)	-9.3
2019	67.0 (66.4-67.6)	51.9 (48.8-55.0)	63.5 (62.3-64.6)	66.2 (64.7-67.8)	69.2 (68.1-70.3)	70.6 (69.4-71.8)	18.7	70.7 (69.9-71.6)	63.7 (62.8-64.5)	-7.0	68.6 (67.5-69.6)	69.1 (68.2-70.1)	62.1 (61.0-63.2)	-6.5
Chile														
2009/10	78.9 (76.7-80.8)	78.1 (73.2-82.3)	80.8 (77.0-84.1)	82.8 (79.6-85.5)	78.8 (71.4-84.8)	69.0 (61.7-75.4)	-9.1	82.7 (79.6-85.4)	75.2 (72.3-77.9)	-7.5	79.4 (75.7-82.6)	79.3 (75.7-82.5)	77.3 (73.4-80.8)	-2.1
2016/17	70.5 (68.0-72.8)	62.3 (54.9-69.1)	70.1 (65.2-74.6)	68.9 (64.4-73.1)	75.4 (70.0-82.2)	74.9 (69.6-79.5)	12.6	77.7 (74.2-80.8)	63.4 (60.0-66.7)	-14.3	74.4 (70.4-78.1)	68.5 (64.0-72.6)	67.5 (63.0-71.7)	-6.9
Peru														
2009/10	78.6 (77.8-79.4)	86.0 (84.4-87.4)	82.0 (80.4-83.5)	74.7 (72.9-76.4)	74.5 (72.1-76.8)	74.4 (72.0-76.7)	-11.6	82.1 (80.8-83.2)	76.0 (74.9-77.1)	-6.1	76.6 (75.2-77.9)	80.6 (79.2-81.9)	79.2 (77.5-80.9)	2.6
2011	69.6 (68.3-70.9)	76.2 (73.6-78.6)	74.0 (71.7-76.3)	66.8 (64.1-69.5)	63.4 (59.7-67.0)	65.9 (62.1-69.4)	-10.3	78.5 (76.5-80.3)	62.8 (61.0-64.6)	-15.7	68.6 (66.5-70.6)	70.9 (68.7-73.0)	69.6 (66.9-72.2)	1.0
Uruguay†														
2006	69.4 (67.2-71.6)	69.6 (63.0-75.5)	72.9 (68.7-76.7)	70.0 (65.4-74.1)	67.8 (62.2-72.9)	66.2 (60.7-71.3)	-3.4	75.0 (71.4-78.3)	64.3 (61.5-67.0)	-10.7	77.4 (73.3-81.1)	65.7 (61.8-69.3)	66.2 (62.6-69.6)	-11.2
2013	79.4 (77.4-81.2)	74.7 (69.8-79.0)	80.7 (76.6-84.2)	79.8 (76.4-82.8)	83.5 (79.2-87.1)	72.2 (62.7-80.1)	-2.5	82.5 (79.5-85.2)	76.4 (73.9-78.8)	-6.1	82.7 (79.5-85.5)	78.4 (74.9-81.5)	74.4 (70.8-77.7)	-8.3
Venezuela														
2014/17	50.5 (47.2-53.8)	51.0 (44.1-57.8)	53.1 (44.9-61.2)	49.0 (42.0-56.0)	51.5 (42.9-60.1)	49.0 (42.2-55.9)	-2.0	57.1 (50.5-63.5)	48.2 (44.4-52.1)	-8.9	58.6 (50.4-66.4)	53.1 (47.6-58.6)	45.7 (40.9-50.5)	-12.9
2018/20	56.8 (53.3-60.2)	56.4 (48.6-63.8)	63.7 (54.8-71.7)	53.9 (46.5-61.1)	58.9 (50.0-67.3)	54.5 (47.5-61.2)	-1.9	65.0 (58.0-71.4)	54.1 (50.1-58.1)	-10.9	62.3 (54.2-69.7)	57.9 (52.4-63.2)	53.1 (47.6-58.5)	-9.2

*Values are presented in percentage (95% CI) and the gap is presented as percentage point. Gap of education refers to Q5 – Q1. Gap of gender refers to Women – Men. Gap of age group refers to 50-64 – 18-34.

†Uruguay 2006 only included adults between 25 and 64 y.

**Table 5:** Temporal trends of sitting time practice (≥8 hours/d) in South American countries*

	Total	Quintiles of education						Gender			Age group			
	**Q1**	**Q2**	**Q3**	**Q4**	**Q5**	**Gap**	**Men**	**Women**	**Gap**	**18-34**	**35-49**	**50-64**	**Gap**
Argentina														
2005	14.0 (13.2-14.8)	6.5 (5.1-8.3)	8.2 (6.8-9.8)	10.9 (9.3-12.8)	12.8 (11.2-14.6)	24.9 (23.2-26.7)	18.4	16.5 (15.3-17.9)	11.6 (10.7-12.6)	-4.9	15.7 (14.5-17.0)	12.5 (11.3-13.8)	12.7 (11.2-14.4)	-3.0
2009	15.2 (14.6-15.9)	10.3 (8.6-12.4)	8.9 (7.7-10.3)	12.7 (11.3-14.2)	13.7 (12.4-15.2)	23.7 (22.2-25.2)	13.4	17.4 (16.4-18.6)	13.2 (12.4-14.1)	-4.2	16.7 (15.6-17.8)	14.0 (13.0-15.2)	14.0 (12.7-15.4)	-2.7
2013	16.8 (15.9-17.6)	13.9 (11.6-16.6)	12.5 (11.1-14.2)	12.6 (10.8-14.7)	15.6 (13.9-17.4)	24.4 (22.7-26.2)	10.5	19.3 (18.0-20.7)	14.5 (13.5-15.5)	-4.8	17.8 (16.4-19.3)	15.8 (14.3-17.4)	16.3 (15.1-17.7)	-1.5
2018	16.3 (15.5-17.2)	8.8 (6.0-12.9)	11.0 (9.2-13.0)	11.1 (9.6-12.7)	13.4 (11.8-15.2)	24.8 (23.2-26.5)	16.0	18.7 (17.4-20.2)	14.1 (13.1-15.2)	-4.6	18.3 (16.9-19.9)	16.1 (14.8-17.5)	12.8 (11.5-14.3)	-5.5
Bolivia†														
2008	10.7 (10.2-11.3)	5.8 (5.1-6.4)	10.5 (8.4-13.0)	10.1 (8.7-11.6)	13.4 (12.1-14.9)	17.8 (16.4-19.3)	12.0	14.3 (13.0-15.7)	9.7 (9.1-10.3)	-4.6	11.4 (10.8-12.2)	9.6 (8.7-10.5)	-	-1.8
2016	9.0 (8.5-9.6)	4.5 (3.0-6.8)	5.5 (4.6-6.5)	7.5 (6.7-8.3)	9.3 (7.4-11.5)	15.7 (14.2-17.3)	11.2	11.3 (10.1-12.6)	8.3 (7.6-9.0)	-3.0	9.2 (8.5-10.0)	8.8 (7.9-9.8)	-	-0.4
Chile														
2009/10	10.8 (9.1-12.8)	5.3 (3.1-9.0)	7.3 (5.0-10.6)	10.2 (7.2-14.3)	11.0 (7.2-16.5)	21.6 (16.0-28.4)	16.3	12.3 (9.6-15.6)	9.4 (7.4-11.8)	-2.9	12.0 (9.2-15.5)	9.0 (6.9-11.7)	11.7 (7.9-16.9)	-0.3
2016/17	11.6 (9.9-13.4)	6.3 (3.1-12.3)	8.1 (5.8-11.1)	10.3 (7.7-13.5)	16.6 (10.9-24.5)	17.6 (13.4-22.7)	11.3	13.4 (10.8-16.5)	9.8 (7.9-12.0)	-3.6	14.5 (11.5-18.2)	10.4 (7.9-13.5)	9.0 (6.7-11.9)	-5.5
Peru														
2009/10	11.7 (11.1-12.3)	6.8 (5.9-7.9)	8.1 (7.2-9.1)	14.9 (13.6-16.3)	13.1 (11.4-14.9)	17.0 (15.2-19.1)	10.2	15.4 (14.4-16.5)	9.0 (8.3-9.7)	-6.4	14.9 (13.9-16.0)	9.8 (8.9-10.8)	9.0 (8.0-10.2)	-5.9
2011	21.6 (20.5-22.7)	15.9 (13.9-18.0)	17.2 (15.5-19.1)	21.7 (19.5-24.0)	25.0 (22.0-28.3)	31.4 (28.1-34.9)	15.5	22.0 (20.3-23.8)	21.3 (19.9-22.7)	-0.7	25.0 (23.2-26.9)	20.2 (18.5-22.1)	18.1 (16.1-20.3)	-6.9
Uruguay‡														
2006	17.6 (15.7-19.6)	7.1 (4.5-11.0)	7.8 (5.5-11.0)	15.3 (12.1-19.1)	20.2 (15.9-25.2)	36.4 (31.0-42.1)	29.3	20.0 (26.9-23.5)	15.4 (13.4-17.6)	-4.6	19.9 (16.2-24.2)	17.0 (14.1-20.3)	16.0 (13.4-19.0)	-3.9
2013	23.1 (21.0-25.3)	14.9 (11.4-19.4)	18.7 (14.9-23.0)	23.2 (19.6-27.2)	37.2 (31.7-43.0)	26.3 (18.8-35.5)	11.4	25.6 (22.3-29.3)	20.7 (18.4-23.3)	-4.9	25.3 (21.8-29.2)	22.4 (19.1-26.0)	19.8 (16.8-23.2)	-5.5
Venezuela														
2014/17	14.4 (12.3-16.9)	8.8 (5.6-13.6)	18.2 (12.6-25.4)	15.3 (10.9-21.1)	14.6 (9.5-21.9)	16.5 (12.0-22.3)	7.7	17.9 (13.4-23.5)	13.3 (10.9-16.1)	-4.6	13.8 (9.0-20.5)	17.6 (13.8-22.2)	12.2 (9.4-15.8)	-1.6
2018/20	7.3 (5.7-9.3)	4.2 (2.0-8.7)	7.3 (3.8-13.4)	5.6 (3.0-10.1)	12.9 (8.0-20.1)	7.9 (4.9-12.6)	3.7	8.6 (5.4-13.5)	6.8 (5.1-9.2)	-1.8	7.9 (4.5-13.5)	6.2 (4.0-9.5)	8.0 (5.5-11.5)	0.1

*Values are presented in percentage (95% CI) and the gap is presented as percentage point. Gap of education refers to Q5-Q1. Gap of gender refers to Women – Men. Gap of age group refers to 50-64 – 18-34 or 35-49 – 18-34 for Bolivia.

†Bolivia only included people between 18 and 49 years.

‡Uruguay 2006 only included adults between 25 and 64 years.

Temporal trends in sitting time are presented in **Table 5**. There was an increasing trend in Argentina (2005 = 14.0% vs 2018 = 16.3%), Peru (2009/2010 = 11.7% vs 2011 = 21.6%) and Uruguay (2006 = 17.6% vs 2013 = 23.1%), while Bolivia (2008 = 10.7% vs 2016 = 9.0%) and Venezuela (2014/2017 = 14.4% vs 2018/2020 = 7.3%) presented a decreasing trend and Chile showed a maintenance. Despite the reducing trend for education inequalities in Uruguay, the inequalities regarding sitting time were relatively constant overtime (**Figure 1**).

## DISCUSSION

We aimed to investigate time trends in different domains of physical activity and sitting time in South America as well as the trends in the inequalities regarding education, gender, and age. Our main findings reveal that leisure-time physical activity increased over the years in most South American countries (in six over the eight countries), but the difference in the prevalence of leisure-time physical activity between the first and fifth quintiles of education also increased over time in most of them as well. The findings for transport physical activity were mixed, with no clear changes in the inequalities over the years. The countries presented a decreasing inequality regarding education for occupational physical activity. Two out of seven countries increased total physical activity, while four increased education inequality and three of them increased age inequality considering the general indicator of total physical activity. Also, no clear patterns of changes in high sitting time over the years were observed and the change regarding its inequality was observed only in Uruguay, which reduced the education inequality.

The increasing trend in leisure-time physical activity, as well as maintenance or reduction in the occupational physical activity, are consistent with findings from high-income countries as well as from individual countries as Brazil [10,19], which can be a marker of a transition in the domain that most contribute to total physical activity [20]. Despite the general trends, there were specific trends considering quintiles of education. For leisure-time physical activity, the highest increases were in the highest quintiles of education, consequently increasing inequalities. These findings are in line with previous research regarding the inequalities in leisure-time physical activity [21], in which the inequalities regarding educational level increased over the years in Brazil.

If countries keep with the same strategies or rely on promoting only leisure-time physical activity as they are doing, they may see some improvements, but at the same time, the public investment will sustain or increase inequalities. A possible explanation for this is the reverse equity hypothesis, in which investments and innovations come first to the most privileged populations and consequently would not be the priority, increasing inequalities [22]. Leisure-time physical activity becomes a reflection of social inequalities in most countries. As people with lower income or educational level are more likely to have inflexible jobs (eg, rigid schedules) and spend more time in passive transport by bus or train as they need to move around cities from peripheral areas to downtown or commercial areas, more tailored strategies are required for these groups as they have reduced time for engaging in physical activity or staying with their families. Countries may promote leisure physical activity by making physical activity more accessible in neighborhoods through public programs in streets and parks, such as the ‘*Ciclovía program*’ in Colombia or the ‘*Academia da Cidade*’ program in Brazil [23,24]. They also have to plan and invest in infrastructure that makes physical activity the easy option when moving around a neighborhood and choosing a place to play or exercise, especially in the most disadvantaged communities [25]. Also, countries should develop specific policies for physical activity and sedentary behavior [26].

The highest decreases or maintenance in occupational physical activity were observed in the lower quintiles of education. Part of the inequalities can be related to the higher levels of occupational physical activity in the group with lower education and possibly, they turned to more sedentary jobs [20]. Although the benefits of occupational physical activity are controversial [27], political willingness is needed for promoting healthier working conditions in all South American countries.

Even with small variations over time, when analyzing the findings for physical activity in transport separately by education quintiles, there were substantial increases in the highest education quintiles in Argentina, Chile, and Peru. In addition, the highest quintiles were the groups that presented the lowest reductions of active transport in Uruguay, as well as are stable in Brazil, while there was a reduction in this behavior in the lowest quintile. Considering that active transport is more practiced among participants with lower education in South American countries [8], there may be a change in active transport culture in this sub-continent, towards a less unequal active transport. A coordination between organizations and governments for promoting cycling and walking for transportation is needed to improve the equality of infrastructure for active transportation. Efforts should not only be focused on facilities or infrastructure, but also speed regulations, speed enforcement cameras, opening streets for pedestrians, while highlighting the potential benefits for local economy and safety for streets users [25,28].

Similarly, we found that the highest increases in occupational physical activity occurred in the highest quintile of education. There are different possibilities for the increase of occupational physical activity. For example, it is possible that the characteristics of the jobs changed over time or even the country passed through an economic crisis that changed the patterns of employment in the population and this should be inferred within the context of each country.

There were minor variations for total physical activity. However, it is worth noting that there was a general trend for increasing the education inequalities in Argentina, Brazil, Chile, and Colombia, possibly indicating that leisure-time physical activity had an important role in the trends over time. Also, it is expected a reduction in the total time of occupational physical activity [20], partly explaining the highest reductions of physical activity in the lowest quintiles of education, which is the group with highest occupational physical activity [8]. We also found a slightly increasing trend for sitting time across the South American countries, with minor changes regarding education, gender, and age inequalities. The increase highlights that despite actions aiming to increase physical activity practice, policies focused on reducing sitting time, especially for leisure-time passive activities such as watching TV, should be formulated, including tackling sitting time in the national policies for health behaviors or physical activity [17,26].

Over time, there were no substantial changes for gender and age inequalities with women practicing lower leisure-time and occupational physical activity and older people presenting lower physical activity in all domains. Gender inequality is a recognized challenge in physical activity research [29,30] and more pronounced public policy actions should be taken to tackle the gender inequalities. Despite the direct actions for increasing physical activity among women as including this in national physical activity plans, other factors may also contribute to reducing gender differences as environmental changes, including the improvement of walkability [31], cycle lanes, access to public transport, and housing density [32].

The present study included more than 550 000 adults from nine out of twelve South American countries to estimate time trends in different domains of physical activity and sitting time in South America, representing approximately 98% of the South American adults, and this is the first study of this kind to our knowledge. However, our findings have limitations that should be considered. First, even though we considered nonzero min/week for the different physical activity domains, there were small variations in the questionnaires in Bolivia, Brazil, Chile, Ecuador, and Peru, which may have changed the findings. However, our focus was on inequalities and there is no plausibility that the changes have affected the inequalities themselves. Second, we interpret inequalities by the differences between the lowest and highest quintiles of education, gender, or between the youngest and the oldest, and possibly a higher prevalence of the indicators can lead to an inflation in the inequalities. However, this method is easy to apply and interpret [33]. Third, despite estimates from Venezuela, trends are based on different surveys in different years rather than individual data. Fourth, the trend for each country covers different periods and different sample sizes and the comparability between the countries should take into consideration the country-specific context of the period (eg, passing through an economic crisis) as it may lead to history bias.

## CONCLUSIONS

Our findings suggest that the total physical activity, leisure-time physical activity, and sitting time increased over the years, while there were minor changes for transport and occupational physical activity, with mixed findings across the countries. The socioeconomic inequalities increased over the years for total and leisure-time physical activity in most countries, while were constant for transport, occupational, and sitting time. Also, the gender, and age inequalities were constant over time, with women and older adults presenting lower total, leisure-time, and occupational physical activity. Future South American countries efforts may be warranted to promote physical activity and reduce sedentary time in adults, while addressing inequalities when implementing actions. Also, a continuous surveillance on physical activity and sitting time levels is essential to evaluate the effectiveness of the current strategies for promoting physical activity and reducing sitting time.

## Additional material


Online Supplementary Document
