# Supplementary material for: Time trends and inequalities of physical activity domains and sitting time in South America
Source: J Glob Health. 2022 Apr 2;12:04027. doi: 10.7189/jogh.12.04027 (PMC8974534; doi:10.7189/jogh.12.04027)
Supplement: Online Supplementary Document [file jogh-12-04027-s001.pdf]

**Table S1.** Details about the sample, design, questionnaires about physical activity and sitting time as well as education categories.

| Survey | Design | Sample | Questionnaire | Domains | Modifications or specifications | Education categories |
|---|---|---|---|---|---|---|
| *Argentina* | | | | | | |
| 2005<br><br>"Encuesta Nacional de Factores de Riesgo" | Stratified four-stage cluster design<br><br>Setting: urban | 34,523<br>(474 missings from the original sample) | IPAQ-short version | Total physical activity and sitting time | None | 1. No education/ primary incomplete<br>2. Primary complete<br>3. Secondary incomplete<br>4. Secondary complete<br>5. Higher/special education |
| 2009<br><br>"Encuesta Nacional de Factores de Riesgo" | Stratified four-stage cluster design<br><br>Setting: urban | 28,763<br>(218 missings from the original sample) | IPAQ-short version | Leisure-time, transport, occupational, total and sitting time | Complementary questions about the practice of physical activity in each domain (yes or not) were included. | 1. No education/ primary incomplete<br>2. Primary complete<br>3. Secondary incomplete<br>4. Secondary complete<br>5. Higher/special education |
| 2013<br><br>"Encuesta Nacional de Factores de Riesgo" | Stratified four-stage cluster design<br><br>Setting: urban | 30,525<br>(1,840 missings from the original sample) | IPAQ-short version | Leisure-time, transport, occupational, total and sitting time | Complementary questions about the practice of physical activity in each domain (yes or not) were included. | 1. No education/ primary incomplete<br>2. Primary complete<br>3. Secondary incomplete<br>4. Secondary complete<br>5. Higher/special education |
| 2018<br><br>"Encuesta Nacional de Factores de Riesgo" | Stratified four-stage cluster design<br><br>Setting: urban | 22,530<br>(985 missings from the original sample) | IPAQ-short version | Leisure-time, transport, occupational, total and sitting time | Complementary questions about the practice of physical activity in each domain (yes or not) were included. | 1. No education/ primary incomplete<br>2. Primary complete<br>3. Secondary incomplete<br>4. Secondary complete<br>5. Higher/special education |
| *Bolivia* | | | | | | |
| 2008<br><br>"Encuesta Nacional de Demografía y Salud" | Stratified two-stage cluster design | 18,997<br>(1,098 missings from the original sample) | Question about leisure-time physical activity and sitting time | Leisure-time and sitting time | N/A | 1. No education, incomplete primary<br>2. Complete primary |

| | | | | | | |
|---|---|---|---|---|---|---|
| | Setting: urban and rural | | | | | 3. Incomplete secondary<br>4. Complete secondary or technical education<br>5. Higher than secondary |
| 2016<br><br>“Encuesta Nacional de Demografía y Salud” | Stratified two-stage cluster design<br><br>Setting: urban and rural | 13,768<br>(902 missings from the original sample) | Question about leisure-time physical activity and sitting time | Leisure-time and sitting time | N/A | 1. No education, incomplete primary<br>2. Complete primary<br>3. Incomplete secondary<br>4. Complete secondary or technical education<br>5. Higher than secondary |
| *Brazil* | | | | | | |
| 2008<br><br>“Pesquisa Nacional por Amostra de Domicílios” | Stratified three-stage cluster design<br><br>Setting: urban and rural | 145,271<br>(2.146 missings from the original sample) | VIGITEL questionnaire | Leisure-time | Did not include the questions about transport and occupational domains | 1. No education<br>2. Primary incomplete<br>3. Primary complete and secondary incomplete<br>4. Secondary complete and higher incomplete<br>5. Higher education complete |
| 2013<br><br>“Pesquisa Nacional de Saúde” | Stratified three-stage cluster design<br><br>Setting: urban and rural | 51,869 | VIGITEL questionnaire | Leisure-time, transport, occupational and total | None | 1. No education<br>2. Primary incomplete<br>3. Primary complete and secondary incomplete<br>4. Secondary complete and higher incomplete<br>5. Higher education complete |
| 2015<br><br>“Pesquisa Nacional por Amostra de Domicílios” | Stratified three-stage cluster design<br><br>Setting: urban and rural | 56,405<br>(2,258 missings from the original sample) | VIGITEL questionnaire | Leisure-time | Did not include the questions about transport and occupational domains | 1. No education<br>2. Primary incomplete<br>3. Primary complete and secondary incomplete<br>4. Secondary complete and |

| | | | | | | |
|---|---|---|---|---|---|---|
| | | | | | | higher incomplete<br>5. Higher education complete |
| 2019<br><br>“Pesquisa Nacional de Saúde” | Stratified three-stage cluster design<br><br>Setting: urban and rural | 71,344<br>(2,854 missings from the original sample) | VIGITEL questionnaire | Leisure-time, transport, occupational and total | None | 1. No education<br>2. Primary incomplete<br>3. Primary complete and secondary incomplete<br>4. Secondary complete and higher incomplete<br>5. Higher education complete |
| *Chile* | | | | | | |
| 2009/10<br><br>“Encuesta Nacional de Salud” | Stratified and multi-stage cluster design<br><br>Setting: urban and rural | 3,993<br>(63 missings from the original sample) | GPAQ | Leisure-time, transport, occupational, total and sitting time | None | Education quintiles created using the number of years of education |
| 2016/17<br><br>“Encuesta Nacional de Salud” | Stratified and multi-stage cluster design<br><br>Setting: urban and rural | 4,249<br>(38 missings from the original sample) | GPAQ | Leisure-time, transport, occupational, total and sitting time | None | Education quintiles created using the number of years of education |
| *Colombia* | | | | | | |
| 2005<br><br>“Encuesta Nacional de la Situación Nutricional” | Three-stage cluster design<br><br>Setting: urban and rural | 12,606<br>(1,995 missings from the original sample) | IPAQ | Leisure-time, transport, and occupational | | Education quintiles created using the number of years of education |
| 2010<br><br>“Encuesta Nacional de la Situación Nutricional” | Three-stage cluster design<br><br>Setting: urban and rural | 14,208<br>(209 missings from the original sample) | IPAQ | Leisure-time and transport | Did not include questions about occupational physical activity | 1. No education<br>2. Incomplete or complete primary<br>3. Complete secondary<br>4. Technical education<br>5. College or higher |

| | | | | | | |
|---|---|---|---|---|---|---|
| 2015<br><br>"Encuesta Nacional de la Situación Nutricional" | Three-stage cluster design<br><br>Setting: urban and rural | 17,788<br>(155 missings from the original sample) | IPAQ | Leisure-time and transport | Did not include questions about occupational physical activity | Education quintiles created using the number of years of education |
| *Ecuador* | | | | | | |
| 2011/12<br><br>"Encuesta Nacional de Salud y Nutrición" | Three-stage cluster design<br><br>Setting: urban and rural | 19,833*<br><br>*with merged data on physical activity and sociodemographic indicators | IPAQ | Leisure-time and transport | Did not include questions about occupational physical activity | Education quintiles created using the number of years of education |
| 2018<br><br>"Encuesta STEPS Ecuador" | Three-stage cluster design<br><br>Setting: urban and rural | 4,341<br>(297 missings from the original sample) | GPAQ | Leisure-time and transport | Included occupational physical activity, but it was not used in the analyzes as occupational physical activity was not included in the 2011/12 survey | Education quintiles created using the number of years of education |
| *Peru* | | | | | | |
| 2007/08<br><br>"Encuesta Nacional de Hogares, Módulo de Mediciones Antropométricas" | Three-stage cluster design<br><br>Setting: urban and rural | 11,511<br>(2,834 missings from the original sample)*<br><br>*considering the 14,345 with merged data on physical activity and sociodemographic factors | Question about leisure-time physical activity | Leisure-time physical activity | None | 1. No education or incomplete primary<br>2. Complete primary or incomplete secondary<br>3. Complete secondary<br>4. Incomplete college or technical<br>5. College or higher |
| 2009/10<br><br>"Encuesta Nacional de Hogares, Módulo de Mediciones Antropométricas" | Three-stage cluster design<br><br>Setting: urban and rural | 20,164<br>(860 missings from the original sample)<br><br>*considering the 21,024 with merged data on physical activity and sociodemographic factors | IPAQ | Leisure-time, transport, occupational, total and sitting time | None | 1. No education or incomplete primary<br>2. Complete primary or incomplete secondary<br>3. Complete secondary<br>4. Incomplete college or technical<br>5. College or higher |

| | | | | | | |
|---|---|---|---|---|---|---|
| 2011<br><br>"Encuesta Nacional de Hogares, Módulo de Mediciones Antropométricas" | Three-stage cluster design<br><br>Setting: urban and rural | 9,013<br>(15 missings from the original sample)<br><br>*considering the 9,028 with merged data on physical activity and sociodemographic factors | IPAQ | Leisure-time, transport, occupational, total and sitting time | None | 1. No education or incomplete primary<br>2. Complete primary or incomplete secondary<br>3. Complete secondary<br>4. Incomplete college or technical<br>5. College or higher |
| *Uruguay* | | | | | | |
| 2006<br><br>"Encuesta Nacional de Factores de Riesgo de Enfermedades Crónicas No Transmisibles" | Three-stage cluster design<br><br>Setting: urban | 1,954<br>(4 missings from the original sample) | GPAQ | Leisure-time, transport, occupational, total and sitting time | None | 1. No education or incomplete primary<br>2. Complete primary or incomplete secondary<br>3. Complete secondary<br>4. Technical<br>5. College or higher |
| 2013<br><br>"Encuesta Nacional de Factores de Riesgo de Enfermedades Crónicas No Transmisibles" | Three-stage cluster design<br><br>Setting: urban | 2,458<br>(174 missings from the original sample) | GPAQ | Leisure-time, transport, occupational, total and sitting time | None | 1. No education or incomplete primary<br>2. Complete primary or incomplete secondary<br>3. Complete secondary<br>4. Technical<br>5. College or higher |
| *Venezuela** | | | | | | |
| 2014/17<br><br>"Estudio Venezoelano de Salud Cardio-Metabólica" | Four-stage cluster design<br><br>Setting: urban and rural | 879<br>(83 missings from the original sample) | IPAQ-short version | Total physical activity and sitting time | None | 1. No education, incomplete primary or complete primary<br>2. Incomplete secondary<br>3. Complete secondary<br>4. Technical or incomplete college.<br>5. College or higher |
| 2018/20<br><br>"Estudio Venezoelano de Salud Cardio-Metabólica" | Four-stage cluster design | 796<br>(68 missings from the original sample) | IPAQ-short version | Total physical activity and sitting time | None | 1. No education, incomplete primary or complete primary |

| | | | | | | |
|---|---|---|---|---|---|---|
| | Setting: urban and rural | | | | | 2. Incomplete secondary<br>3. Complete secondary<br>4. Technical or incomplete college.<br>5. College or higher |

Note. *The *Estudio Venezoelano de Salud Cardio-Metabólica* is a longitudinal study.
